# Comparative genomics of the genus *Roseburia* reveals divergent biosynthetic pathways that may influence colonic competition among species

**DOI:** 10.1099/mgen.0.000399

**Published:** 2020-06-26

**Authors:** Ethan T. Hillman, Ariangela J. Kozik, Casey A. Hooker, John L. Burnett, Yoojung Heo, Violet A. Kiesel, Clayton J. Nevins, Jordan M.K.I. Oshiro, Melissa M. Robins, Riya D. Thakkar, Sophie Tongyu Wu, Stephen R. Lindemann

**Affiliations:** ^1^​ Department of Agricultural and Biological Engineering, Purdue University, West Lafayette, IN 47907, USA; ^2^​ Purdue University Interdisciplinary Life Science Program (PULSe), Purdue University, West Lafayette, IN 47907, USA; ^3^​ Department of Comparative Pathobiology, Purdue University, West Lafayette, IN 47907, USA; ^4^​ Department of Food Science, Purdue University, West Lafayette, IN 47907, USA; ^5^​ Department of Agronomy, Purdue University, West Lafayette, IN 47907, USA; ^6^​ Department of Nutrition Science, Purdue University, West Lafayette, IN 47907, USA; ^7^​ Whistler Center for Carbohydrate Research, Purdue University, West Lafayette, IN 47907, USA; ^†^​ Present address: Division of Pulmonary and Critical Care Medicine, Department of Internal Medicine, University of Michigan, Ann Arbor, MI 48109, USA; ^‡^​ Present address: Department of Soil and Water Sciences, University of Florida, Gainesville, FL 32603, USA

**Keywords:** *Lachnospiraceae*, *Roseburia*, comparative genomics, B vitamin biosynthesis, amino acid biosynthesis, butyrate synthesis

## Abstract

*
Roseburia* species are important denizens of the human gut microbiome that ferment complex polysaccharides to butyrate as a terminal fermentation product, which influences human physiology and serves as an energy source for colonocytes. Previous comparative genomics analyses of the genus *Roseburia* have examined polysaccharide degradation genes. Here, we characterize the core and pangenomes of the genus *Roseburia* with respect to central carbon and energy metabolism, as well as biosynthesis of amino acids and B vitamins using orthology-based methods, uncovering significant differences among species in their biosynthetic capacities. Variation in gene content among *Roseburia* species and strains was most significant for cofactor biosynthesis. Unlike all other species of *Roseburia* that we analysed, *Roseburia inulinivorans* strains lacked biosynthetic genes for riboflavin or pantothenate but possessed folate biosynthesis genes. Differences in gene content for B vitamin synthesis were matched with differences in putative salvage and synthesis strategies among species. For example, we observed extended biotin salvage capabilities in *R. intestinalis* strains, which further suggest that B vitamin acquisition strategies may impact fitness in the gut ecosystem. As differences in the functional potential to synthesize components of biomass (e.g. amino acids, vitamins) can drive interspecies interactions, variation in auxotrophies of the *Roseburia* spp. genomes may influence *in vivo* gut ecology. This study serves to advance our understanding of the potential metabolic interactions that influence the ecology of *Roseburia* spp. and, ultimately, may provide a basis for rational strategies to manipulate the abundances of these species.

## Data Summary

The authors confirm that all supporting data, code and protocols have been provided within the article or through supplementary data files. The genomes used for annotation of strains were taken from the following GenBank files: *Roseburia faecis* 2789STDY5608863 (GCA_001405615.1), *R. faecis* M72 (GCA_001406815.1), *Roseburia* sp. 831b (GCA_001940165.1), *Roseburia inulinivorans* 2789STDY5608887 (GCA_001405535.1), *R. inulinivorans* DSM 16841 (GCA_000174195.1), *R. inulinivorans* LI-83 (GCA_001406855.1), *Roseburia intestinalis* LI-82 (GCA_000156535.1), *R. intestinalis* M50/1 (GCA_000209995.1), *R. intestinalis* XB6B4 (GCA_000210655.1), *Roseburia hominis* A2-183 (GCA_000225345.1). All DNA and amino acid sequences, as well as RAST annotations, are provided in the supplemental Excel file in Table S2. Compiled and scored pathway information has been curated in Table S5. Additional scripts and output files can be found on our GitHub repository (https://github.com/ehillman26/Comparative-Genomics-of-Roseburia.git).

**Impact Statement**

Here, we employ a comparative genomics approach to define the core and pangenomes of the genus *Roseburia* and identify species- and strain-level traits that might play a role in their ability to colonize the gut ecosystem. By evaluating *Roseburia *’s proposed physiological and biosynthetic capabilities, we propose underlying principles that may govern the ecology and establishment of specific species and strains. While some of these aspects, such as *Roseburia *’s fermentative end products, have been studied in great detail by other groups, this study connects those findings through a genomic lens and identifies the associated genes across the genus. Our results suggest that B vitamin biosynthesis genes in *Roseburia* spp. might play a large role in their ecology in the gut environment. We present several testable hypotheses that may help unravel the complex nature of these important gut microbes. This understanding may, ultimately, lead to therapeutic approaches that can selectively modulate the gut microbiome.

## Introduction

*Roseburia,* a member of the *Clostridium coccodis* cluster of the phylum Firmicutes [1], is a genus of anaerobic, rod-shaped, Gram-positive bacteria [2]. Species of *Roseburia* are known to be important denizens of the human gut microbiome, with relative abundances estimated at 5–15 % between *Roseburia* spp. and their near neighbours in the genus *Agathobacter* [3] (previously *Eubacterium* [4]); many have been isolated from human faeces [5–7]. *Roseburia* spp. are known to ferment complex polysaccharides entering the colon to butyrate as a terminal product [2, 8]. Butyrate is the preferred energy source of colonocytes in the human large intestine as well as a known histone deacetylase inhibitor [9] and immunomodulatory signal [10]. Recently, it has been suggested that butyrate production by gut microbes and, specifically, *Roseburia* spp., may confer health benefits to humans, including prevention of type II diabetes [11], ulcerative colitis [12] and colon cancer [13, 14]. The abundance of *Roseburia* spp. in faecal samples may also serve as a biomarker for symptomatic pathologies or certain species may be delivered as probiotics for restoration of the gut ecosystem [2, 14–17].

Although several strains within the genus *Roseburia* have been sequenced, there has been no large-scale effort to identify the central catabolic and anabolic genes that compose the core and pangenomes of genus *Roseburia *. As differences in the functional potential to synthesize components of biomass (e.g. amino acids, vitamins) can drive interspecies interactions [18] due, in part, to competition for available ‘public good’ nutrients in communities [19], variation in the genomes of *Roseburia* spp. may influence the ecology of these species. This may be especially important for the synthesis of required B vitamin cofactors, as colonic competition for these resources (e.g. vitamin B_12_ and other corrinoids) has been proposed to structure microbiomes [20]. Such competition may arise due to the commonality of B_12_ auxotrophies and its role in essential processes (e.g. deoxyribonucleotide production [21]), as well as efficient host scavenging processes [22] and lability to gastric degradation [23]. Recent comparison of complex carbohydrate metabolism in members of the genus *Roseburia* and their near neighbour *Agathobacter rectalis* (previously *Eubacterium rectale *) revealed extensive niche partitioning of these species around polysaccharide utilization [24]. By pairing genomic prediction and experimental evidence, Sheridan *et al*. determined that carbohydrate substrate preferences with respect to tested oligo- and polysaccharides were relatively stable at the species and, to an extent, the genus level, with some strain-level differentiation. Despite some divergences in carbohydrate preferences, these organisms share their core fermentative metabolisms, containing highly syntenic operons with the genes required for butyrogenesis from pyruvate [8, 25–27].

In this study, we aimed to (1) define the core and pangenome of the genus *Roseburia* with respect to central carbon and energy metabolism and biosynthetic genes and (2) evaluate the degree to which species- and strain-level differences in auxotrophies might influence competition among these organisms in the colonic ecosystem. We focused in particular on the mechanisms by which *Roseburia* spp. produce amino acids and B vitamins. B vitamins, including thiamine, riboflavin, niacin, pantothenate, pyridoxine, biotin, folate and cobalamin, are nutrients that serve as coenzymes for reactions in bacterial and eukaryotic cells alike, and alterations in the microbial production of these molecules may influence host health [28]. B vitamins are required cofactors for many central metabolic pathways and are involved in diverse biosynthetic processes. Additionally, derivatives of B vitamins, including niacin and riboflavin, also play a role in maintaining cellular oxidative balance. Lack of genes required for biosynthesis of required amino acids and vitamins in certain *Roseburia* strains would require that they successfully compete with the human host and other gut species for these nutrients in the colon. Understanding the metabolic interactions that influence the ecology of *Roseburia* spp. may provide mechanistic bases for rational strategies to increase or maintain abundances of these species that may synergize with approaches that employ differing carbohydrate preferences.

## Methods

We examined 11 different genomes from the genus *Roseburia* in this study for comparative genomic analysis, including representatives from all published species with sequenced genomes and genomes unattributed to any species (* Roseburia intestinalis* XB6B4, *R. intestinalis* LI-82, *R. intestinalis* M50/1, *Roseburia hominis* A2-183, *Roseburia inulinivorans* LI-83, *R. inulinivorans* DSM 16841, *R. inulinivorans* 2789STD5608887, *Roseburia faecis* M72, *R. faecis* 2789STDY5608863, *Roseburia* sp. 499, and *Roseburia* sp. 831b). To increase the number of genomes from poorly represented species of the genus *Roseburia* (*n*<3), we also included genomes produced by a high-throughput cultivation of faecal microbiota [29] that displayed high completeness (>95 %) and a lack of obvious contamination (score of ~2 % or lower; see Table S1, available in the online version of this article) using a set of 420 single-copy *Lachnospiraceae* genes as evaluated by the CheckM tool v. 1.0.18 [30] in KBase [31]. To provide consistency in gene modelling and annotation approaches across genomes, each genome was downloaded from the National Center for Biotechnology Information (NCBI) as nucleic acid FASTA files (FNA). Gene models and draft annotation using the SEED were produced by uploading the FNA files to the Rapid Annotation using Subsystem Technology (RAST) version 2.0 web server [32–34] using the normal bacterial translation table, the RAST gene calling algorithm and the ‘Classic RAST’ annotation scheme, which resulted in FIGfams output [35] (see Table S2). The predicted protein FASTA files (FAA) generated by RAST were used for the rest of the annotation approaches. To provide multiple independent functional predictions using hidden Markov model (HMM)-based approaches, the RAST-generated FAA files were examined using the hmmsearch algorithm within HMMER v. 3.1b2 (hmmer.org [36]) and the TIGRFAMs_14.0 and Pfam-A v. 31.0 profile HMM libraries using the provided, HMM-specific trusted cutoffs to generate hits to TIGRFAMs [37] and Pfams [38], respectively. Finally, each FASTA file was uploaded to BlastKOALA (version 2.1), a web annotation service hosted by the Kyoto Encyclopedia of Genes and Genomes (KEGG) [39]. KOALA (KEGG Orthology and Links Annotation) analyses user data by blast using an SSEARCH computation model to assign KO numbers (denoting orthologue groups associated with specific metabolic reactions) to user data [39]. Release 81.0 of the encyclopedia was used for our genome annotations. To evaluate the presence or absence of metabolic pathways, predicted annotations were visualized using KEGGs’s reconstruction pathway mapper and the SEED Genome Browser tool. Orthologue and paralogue tables were generated from RAST FAA files using the parallel orthologue prediction tool PorthoMCL [40] on a local machine. To compare the orthologue output across different strains and define the core *Roseburia* genome, Python (version 3.7.2) scripts were created to count the co-occurrences of orthologous genes across strains (https://github.com/ehillman26/Comparative-Genomics-of-Roseburia.git). For example, if all species in a given comparison (three *R *. *intestinalis* species vs three *R *. *inulinivorans* species) contained the orthologue, then a 1 was added to the sum of total common orthologues for these two species; this logic was repeated for each entry in the orthologue table to determine the number of shared orthologues within each species, between each species, and in the genus *Roseburia *.

Predicted annotations were curated from the integrated output of each annotation tool (TIGRFAMs, FIGfams and KOALA) for each predicted open reading frame, cross-referenced using the RAST-provided locus tags. Domain-based hits (Pfams) were used to propose genes that might fill holes in metabolic pathways that were not identified using other annotation tools and to validate the output of other annotation tools. To reconcile the output of the multiple annotation approaches, we constructed an operational confidence scale that emphasized expert curation (TIGRFAMs) and genome context (Table S5).

### Phylogenetic/phylogenomic tree reconstruction

We reconstructed the phylogeny of the genus *Roseburia* in comparison to other type strains of species within the family *Lachnospiraceae* available with GenBank (https://www.ncbi.nlm.nih.gov/nuccore/). As only a subset of *Roseburia* near neighbours have been sequenced, we initially constructed phylogenetic trees based on full-length 16S rRNA gene sequences. The 16S rRNA gene alignment was created with mega7 [41] using clustal W [42] for multiple sequence alignment; phylogeny was reconstructed using the maximum-likelihood method using the Tamura–Nei substitution model [43] with 1000 bootstraps. For genome-sequenced near neighbours, we also examined phylogenomic relationships using a subset of highly conserved, single-copy genes from the AMPHORA2 database [44], prioritizing type strains of near-neighbour genera and species for inclusion. For phylogenomic reconstruction, we selected genes within the Amphora collection for which TIGRFAM HMMs existed, yielding 18 proteins for concatenation: RpoB (TIGR02013), InfC (TIGR00168), NusA (TIGR01953), RplA (TIGR01169), RplB (TIGR01171), RplD (TIGR03953), RplM (TIGR01066), RplN (TIGR01067), RplP (TIGR01164), RplS (TIGR01024), RplT (TIGR01032), RpsB (TIGR01011), RpsC (TIGR01009), RpsE (TIGR01021), RpsJ (TIGR01049), RpsS (TIGR01050), SmpB (TIGR00086) and Tsf (TIGR00116). Proteins exceeding each of the TIGRFAM HMM trusted cutoffs for each protein within a genome were concatenated; this approach identified a single protein matching each HMM for all genomes. *Prevotella brevis* (as an outgroup) and protein sequences of neighbouring organisms within *Lachnospiraceae* were obtained from GenBank (Table S4). The concatenated amino acid sequences were aligned with clustal W in mega 7, and the maximum-likelihood method was used to construct a tree using the Poisson substitution model [45] for the alignment with 100 bootstraps.

## Results and Discussion

### Phylogenomic analysis reveals that *Roseburia* sp. 499 does not cluster within the genus *Roseburia*

**Fig. 1.**
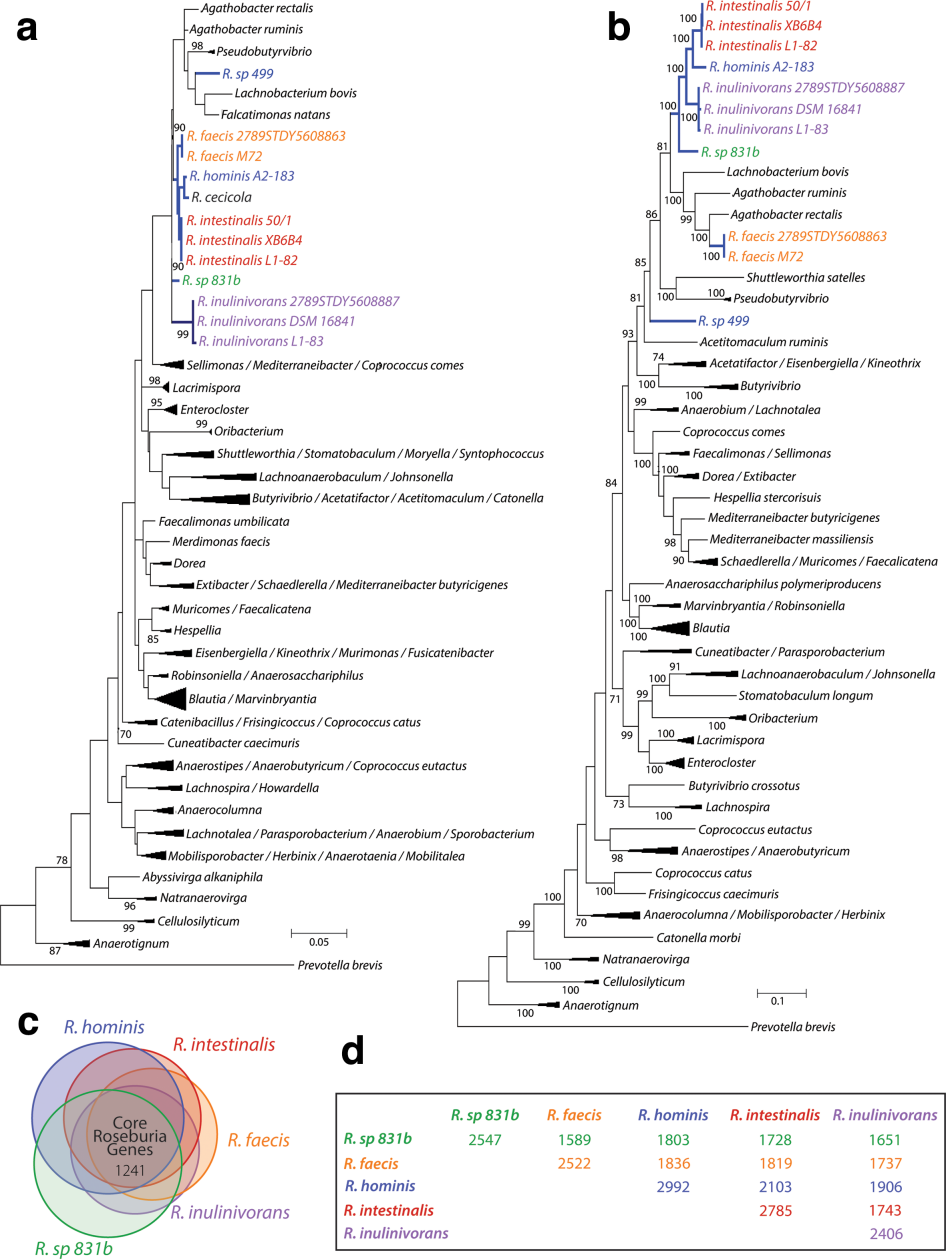
Phylogenetic trees of the family *Lachnospiraceae *. (a) Full-length 16S rRNA gene maximum-likelihood tree from 1000 bootstrap replicates is shown. Genera other than *Roseburia* were collapsed to simplify visualization of the tree. The fully expanded tree including accession numbers can be found in Fig. S1. (b) The 18-gene concatenated maximum-likelihood tree from 100 bootstrap replicates is shown with collapsed nodes and the fully expanded tree can be found in Fig. S2. (Bootstrap scores >70 are reported). (c) *Roseburia* core genome(s) displaying the overlapping orthologous protein encoding genes among the species evaluated. (d) Pairwise comparison of orthologies among *Roseburia* sp. Rows are coloured according to the species and numbers along the diagonal represent the core genome for a given species.

Despite its present tentative taxonomic assignment, 16S rRNA gene-based phylogenetic analysis across the family *Lachnospiraceae* revealed that *Roseburia* sp. 499 did not cluster within a distinct clade formed by other species of the genus *Roseburia *. This species’ 16S rRNA gene sequence instead clustered with 16S rRNA genes from *Pseudobutyrivibrio *, albeit with relatively low bootstrap support (Fig. 1A). This species was originally isolated from swine and was proposed to be a *Roseburia* species in 2013 [46]; however, further physiological and chemotaxonomic analyses of this isolate were never reported to confirm this placement. Unfortunately, the relatively small number of validly published strains within *Lachnospiraceae *, and therefore confirmed 16S rRNA gene sequences, leaves gaps in our understanding of this family [29] and generates uncertainty in taxonomic placement of new isolates. Our results in Fig. 1b suggested that, based upon 16S rRNA gene-based phylogenetics, *R*. sp. 499 might be more appropriately classified as a member of *Pseudobutyrivibrio* rather than *Roseburia *.

To resolve the phylogenetic placement of *R*. sp. 499, we employed a whole-genome approach using concatenated single-copy proteins [47] from each organism, which provided higher resolution and increased confidence in the branches. When curating the genes included in AMPHORA, Wu and Eisen noted that this increased confidence is a result of the conservation of protein-coding genes at the amino acid level rather than the DNA level, where compositional biases in small subunit rRNA exist [48–50]. The concatenated tree (Fig. 1b) agreed with the conclusion of the 16S rRNA gene analysis in placing *Roseburia* sp. 499 well outside the *Roseburia* clade. Interestingly, the concatenated tree also revealed that *R. faecis* clustered with high bootstrap support with *A. rectalis* instead of within the genus *Roseburia *, despite well-supported clustering of the 16S rRNA genes of both *R. faecis* strains within *Roseburia. Eubacterium rectale*, long known to be a physiologically similar near neighbour of *Roseburia *, was recently reclassified as *A. rectalis* based upon its phylogenetic relationships with a newly isolated species [3]. Recent analysis of the carbohydrate-active enzymes of *A. rectalis* and *Roseburia* strains revealed that *R. faecis* M72 GH13 family glycoside hydrolases clustered nearly uniformly with those of the then *E. rectale* strains ATCC 33656, AI-86, M104/1 and T1-815 and separately from other *Roseburia* species [24]. Taken together, the sequence similarities between *R. faecis* and *E. rectale/A. rectalis* suggest that *R. faecis* may be more related to members of *Agathobacter* than to those of *Roseburia *. Because the genus *Eubacterium* is not monophyletic, efforts are presently underway to improve the taxonomy of this group [3]; the genomic evidence presented here suggests that such efforts should also include the genus *Roseburia *. It should be noted that we only considered isolate genomes attributed to *Roseburia* for which the original source of the organism was clear, linking our analysis strongly to the described taxonomy of the genus. Thus, this limitation in the genomes considered restricts our conclusions to the sequenced, current members of *Roseburia *; future expansion in the genus either through isolation, incorporation of metagenome-assembled genomes, or transfer of other members of *Lachnospiraceae* may substantially alter the conclusions drawn here.

### Unifying metabolic properties of the genus *Roseburia*

Using a standardized gene modelling and annotation approach across all *Roseburia* genomes, we identified the core central metabolism and anabolic pathways of the genus. We further aimed to identify differences among strains that might affect their ecology in the human colon. As we aimed to characterize the genomic properties of the genus, we omitted *R*. sp. 499 from further analysis based upon the phylogenomic result that it diverges significantly from the *Roseburia* clade. As their phylogenetic positions were uncertain, we retained strains of *R. faecis* and *R.* sp*.* 831b in further analyses. Using these species as the core members of the genus *Roseburia *, our orthology-based method identified 1241 orthologues that make up the core *Roseburia* genome (Fig. 1c, Table S3). Unsurprisingly, *R. sp*. 831b and *R. faecis* had the lowest number of shared orthologues with respect to the other core *Roseburia* members (Fig. 1d). This finding corroborates the idea that *R. hominis *, *R. intestinalis *, and *R. inulinivorans* species are more closely related, although additional biochemical characterization of *R.* sp. 831b and *R. faecis* may be needed to validate their placement in the genus *Roseburia *.

#### Carbohydrate, central carbon and energy metabolism

**Fig. 2.**
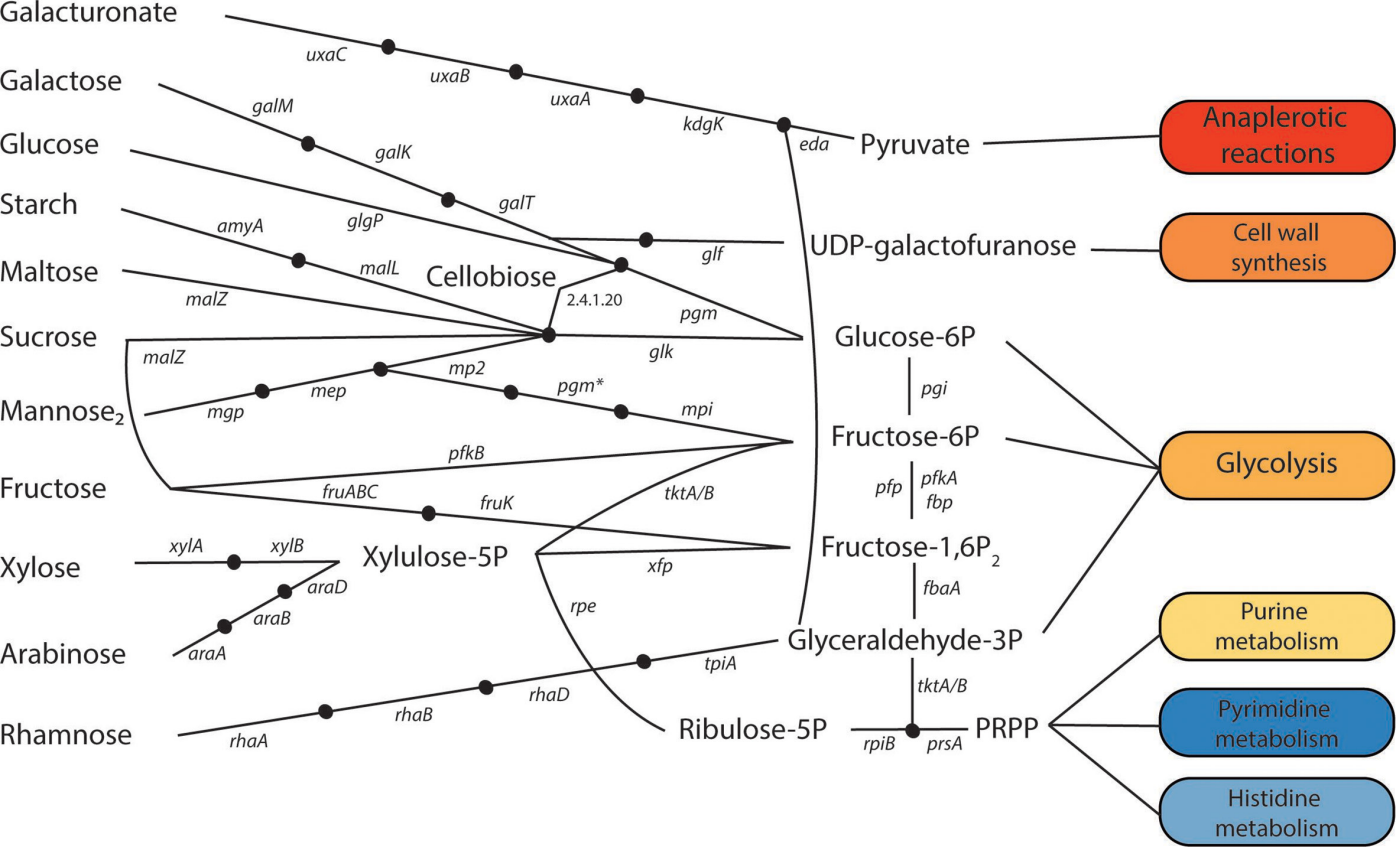
Metabolic pathways of various carbohydrate mono-, di-, and poly-saccharides in *Roseburia *. Each node represents an intermediate compound and each oval represents the metabolic pathway that the metabolite(s) are shuttled to during metabolism. Cofactors are not shown. *uxaC*, glucuronate isomerase; *uxaB*, tagaturonate reductase; *uxaA*, altronate hydrolase; *kdgK*, 2-dehydro-3-deoxygluconokinase; *eda*, 2-dehydro-3-deoxyphosphogluconate aldolase/(4S)-4-hydroxy-2-oxoglutarate aldolase; *galM*, aldose 1-epimerase; *galK*, galactokinase; *galT*=UDPglucose-hexose-1-phosphate uridylyltransferase; *glf*, UDP-galactopyranose mutase, *pgm*, phosphoglucomutase; *glgP*, glycogen phosphorylase; *amyA*, alpha-amylase; *malL*, oligo-1,6-glucosidase; *malZ*, alpha-glucosidase; *glk*, glucokinase; *mgp*, beta-1,4-mannooligosaccharide phosphorylase; *mep*, mannobiose 2-epimerase; *mp2*, 4-O-beta-d-mannosyl-d-glucose phosphorylase; pgm*, bifunctional phosphoglucomutase/phosphomannomutase; *mpi*, mannose 6-phosphate isomerase; *pfkB*, 6-phosphofructokinase 2; *fruA*, PTS fructose-specific enzyme IIABC component; *fruK*, 1-phosphofructokinase; *xylA*, xylose isomerase; *xylB*, xylulokinase; *araA*, L-arabinose isomerase; *araB*, L-ribulokinase; *araD*, L-ribulose-5-phosphate 4-epimerase; *rhaA*, L-rhamnose isomerase; *rhaB*, rhamnulokinase; *rhaD*, rhamnulose-1-phosphate aldolase; *tpiA*, triosephosphate isomerase; *tktA/B*, transketolase 1/2; *xfp*, xylulose-5-phosphate/fructose-6-phosphate phosphoketolase; *rpe*, ribulose-phosphate 3-epimerase; *pgi*, glucose-6-phosphate isomerase; *pfp*, diphosphate-dependent phosphofructokinase; *pfkA*, 6-phosphofructokinase 1; *fbp*, fructose-1,6-bisphosphatase I; *fbaA*, fructose-1,6-bisphosphate aldolase; *rpiB*, ribose 5-phosphate isomerase B; *prsA*, ribose-phosphate pyrophosphokinase; mannose_2_, mannobiose; glucose-6*P*, glucose-6 phosphate; fructose-1,6P_2_, fructose-6 phosphate; fructose-1,6P_2_, fructose-1,6 bisphosphate; glyceraldehyde-3*P*, glyceraldehyde-3 phosphate; riboulose-5*P*, ribulose 5-phosphate.

All *Roseburia* spp. possess the Embden–Meyerhof–Parnas (EMP) glycolytic pathway, which converts glucose to pyruvate. Although we observed 6-phosphogluconolaconase (which converts glucono-1,5-lactone-6-P to 6-phosphogluconate) in all *Roseburia* genomes except those of *R. faecis *, no *Roseburia* spp. encodes the glucose-6-phosphate dehydrogenase required to generate 6-phosphogluconate from glucose-6-P and complete the oxidative branch of the pentose phosphate pathway [51] to ribulose-5-P. Furthermore, we did not detect the genes required to convert 6-phosphogluconate into either 2-keto-3-deoxy-6-phosphogluconate via the Entner–Doudoroff (ED) glycolytic pathway. This finding is consistent with those of most anaerobes (such as *Bifidobacterium* [52]), as the ED pathway yields less ATP per glucose and may be energetically unsustainable in fermentative anaerobes; only ~3 % of strict anaerobes contain the genes for the ED pathway, while 29 % of facultative anaerobes contain the ED pathway or both the EMP and ED pathways [53]. All *Roseburia* genomes, however, displayed evidence of many genes involved in pentose interconversions and conversion of d-fructose-6-P to glyceraldehyde-3-P. However, all genes of the non-oxidative pentose phosphate pathway were present in all species, suggesting that *Roseburia* spp. can convert fructose-6P to ribose-5P. PRPP (5-phosphoribosyl diphosphate) can then be derived from ribose-5P and shuttled to purine, pyrimidine, or histidine biosynthesis (see Fig. 2).

Interestingly, despite the known ability of various *Roseburia* spp. (especially *R. intestinalis *) to ferment monomeric pentoses (e.g. xylose, arabinose) and to consume various xylooligosaccharides, arabinoxylans and arabinogalactans as carbon sources for growth [5, 24], we were unable to detect with high confidence many of the carbohydrate metabolism enzymes required for xylose and arabinose consumption via our approach, particularly in *R. inulinivorans *. However, we did find FIGfam evidence for many of these genes and in many cases these FIGfam calls were found with highly conserved genomic context that was consistent across the strains and the majority of species. For example, although *R. intestinalis* strains all displayed the l-arabinose isomerase required to convert the l-arabinose in arabinoxylan to l-ribulose (*araA*), we could not identify genes involved in phosphorylation of ribulose for conversion into d-xylulose-5-P with TIGRFAMs or KOGs (*araB* or *araD*). Examination of the surrounding gene neighbourhood, however, revealed FIGfam calls for these genes. We consider it likely that known representatives of these genes from *Lachnospiraceae* are lacking within the TIGRFAM and KO reference databases, making their algorithmic identification difficult.

From our analysis of simple carbohydrate metabolism, we found genomic evidence suggesting that all members of *Roseburia* except *R. faecis* and *R.* sp. 831b can utilize galacturonic acid. Our analysis also suggests that all *Roseburia* spp. are able to metabolize glucose, galactose, maltose and sucrose, where only *R. inulinivorans* is likely unable to metabolize xylose, mannose and arabinose. Although all *Roseburia* spp. are missing both the mannose isomerase that converts mannose to fructose and the mannokinase that phosphorylates mannose to mannose-6P as prescribed in the KEGG pathway, a different pathway has been characterized previously from *R. intestinalis* L1-82 [54]. Like other carbohydrate degradation pathways in *Roseburia* spp., the genes for mannose degradation and utilization are organized in an operon including a transcriptional regulator, the associated glucosidases, and an ATP-binding cassette (ABC) transport complex. This mannose degradation and utilization pathway, which is similar to that in *Ruminococcus albus* [55], converts mannobiose with two synergistic mannoside phosphorylases and a mannose epimerase into mannose-1P. Ultimately, a promiscuous phosphoglucomutase/phosphomannosemutase and a bifunctional glucosidase/phosphomannose isomerase convert this to mannose-6P and fructose-6P, respectively, before it enters glycolysis. The fact that this experimentally validated pathway was not captured by BlastKOALA models points to a need for increased coverage within this phylogenetic region.

We also found variation in the different oligosaccharide phosphorylases present among species and strains of *Roseburia *. From our analysis, all *Roseburia* species possess phosphorylases that cleave glycogen, cellobiose, and lacto-N-biose into their respective monosaccharides. These enzymes allow microbes to increase net ATP production by utilizing free orthophosphates to generate phosphosugars instead of consuming ATP to generate them; thus, this may be an advantageous energy efficiency strategy. Interestingly, only the strains of *R. faecis *, two strains of *R. intestinalis* (XB6B4 and LI-82) and *R. inulinivorans* (LI-83) possess a sucrose phosphorylase, while all species contain the *malZ* gene for cleaving sucrose to d-fructose and d-glucose. The *malZ* gene, which is present in all *Roseburia* species, also cleaves maltose into two d-glucose monomers. However, *R. intestinallis* strains LI-82 and M50/1, as well as the *R. inulinivorans* strains 2789STD5608887 and LI-83, have a maltose/trehalose phosphorylase that perform a similar cleavage of maltose to d-glucose and beta-d-glucose 1-phosphate. These phosphorylases are only supported by FIGfam identification and often can act on several similar substrates, which makes these phosphorylases and the *malZ* gene interesting candidates for future genetic and biochemical studies. Other phosphorylases, such as chitobiose phosphorylase (E.C. 2.4.1.280), are present in all *Roseburia* species except *R. inulinivorans* strains 2789STD5608887 and DSM 16841; however, these were also only supported with moderate confidence and should be further characterized. Ultimately, differences in carbohydrate availability from host diets intersecting with different carbohydrate utilization machinery may be a major driver of interspecies competition among *Roseburia* spp. ecology and determine *R. intestinalis* occurrence and abundance patterns in human microbiomes [54, 56].

Interestingly, within *R. inulinivorans* our analysis predicted subspecies-level variation in the ability to use arabinose, as the strain DSM 16841 possesses the same conserved arabinose utilization pathway as the other *Roseburia* species, but the other two *inulinivorans* strains lack it. This finding is particularly interesting because *R. inulinivorans* was previously described as being unable to utilize arabinose for growth [5] and Sheridan and coworkers also found that this strain was unable to grow on arabinoxylan [24]; these phenotypes may, in fact, vary within the species. Similarly, they found that *R. hominis* also does not grow on arabinoxylan; however, the species description [5] indicates that arabinose can be utilized for growth, which agrees with our prediction. Our predictions for *hominis* also include the ability to utilize galactose and galacturonic acid, although the growth of *R. hominis* on galacto-oligosaccharides was not previously observed (pectin, a significant source of galacturonic acid, was not tested). Our predictions match previous descriptions of *R. intestinalis* carbohydrate metabolism [7, 24] for all examined genomes. Likewise, our predictions for *R. faecis* are similar to previous experimental results [24] with respect to arabinose utilization (galacturonic acid, which we predict to not be utilized, was not tested). Sheridan and coworkers also observed mild growth on arabinoxylan and the *araC* gene in a conserved gene neighbourhood with arabinoxylan CAZymes. Further experimental investigation of *Roseburia* species- and strain-specific carbohydrate utilization will be needed to clarify these phenotypic discrepancies in the utilization of dietary fibres and their constituent sugars, as *Roseburia* spp. colonization is dependent upon the dietary fibre intake of the host [57–59].

With respect to energy generation from carbohydrates, all *Roseburia* genomes displayed the genetic capacity for conversion of pyruvate to acetyl-CoA, condensation of acetyl-CoA with oxaloacetate into citrate and conversion of citrate into α-ketoglutarate. However, all *Roseburia* spp. lack both the α-ketoglutarate dehydrogenase complex and α-ketoglutarate synthase and, therefore, cannot interconvert α-ketoglutarate and succinyl-CoA. These enzymes are likely retained for anapleurotic reactions; α-ketoglutarate is a precursor to synthesis of many amino acids, such as glutamate. With respect to the rest of the canonical TCA cycle genes, all genomes contained fumarate hydratase, allowing interconversion of fumarate and malate. *Roseburia* spp. lack the canonical respiratory electron transport and oxidative phosphorylation apparatus; NADH is oxidized as lactate, propionate and butyrate are produced, regenerating oxidized electron carriers. All species encode an F-type ATPase (ATP-forming) that potentially allows utilization of proton motive force generated by excretion of organic acids for ATP generation and pH regulation by balancing H^+^ flux across the membrane [60]. Interestingly, we also detected genes for the classically eukaryotic V/A-type ATPase, which was found (with KO and FIGfam support) exclusively in the *R. intestinalis* genomes [61]. These ATPases are typically used by eukaryotes to acidify vacuoles and consume energy from ATP to export protons [62], although they have also been found in the enterococci to pump cations such as sodium and potassium [63]. This adaptation may arise from gene transfer [64] through a long history of association with eukaryotes and archaea and may grant *R. intestinalis* additional resistance to low pH compared with other members of the genus *Roseburia* [64]. Similar systems are also found in diverse members of the orders Clostridiales and Bacteroidales [65], suggesting that such systems may be important for colonization of the human colon.

**Fig. 3.**
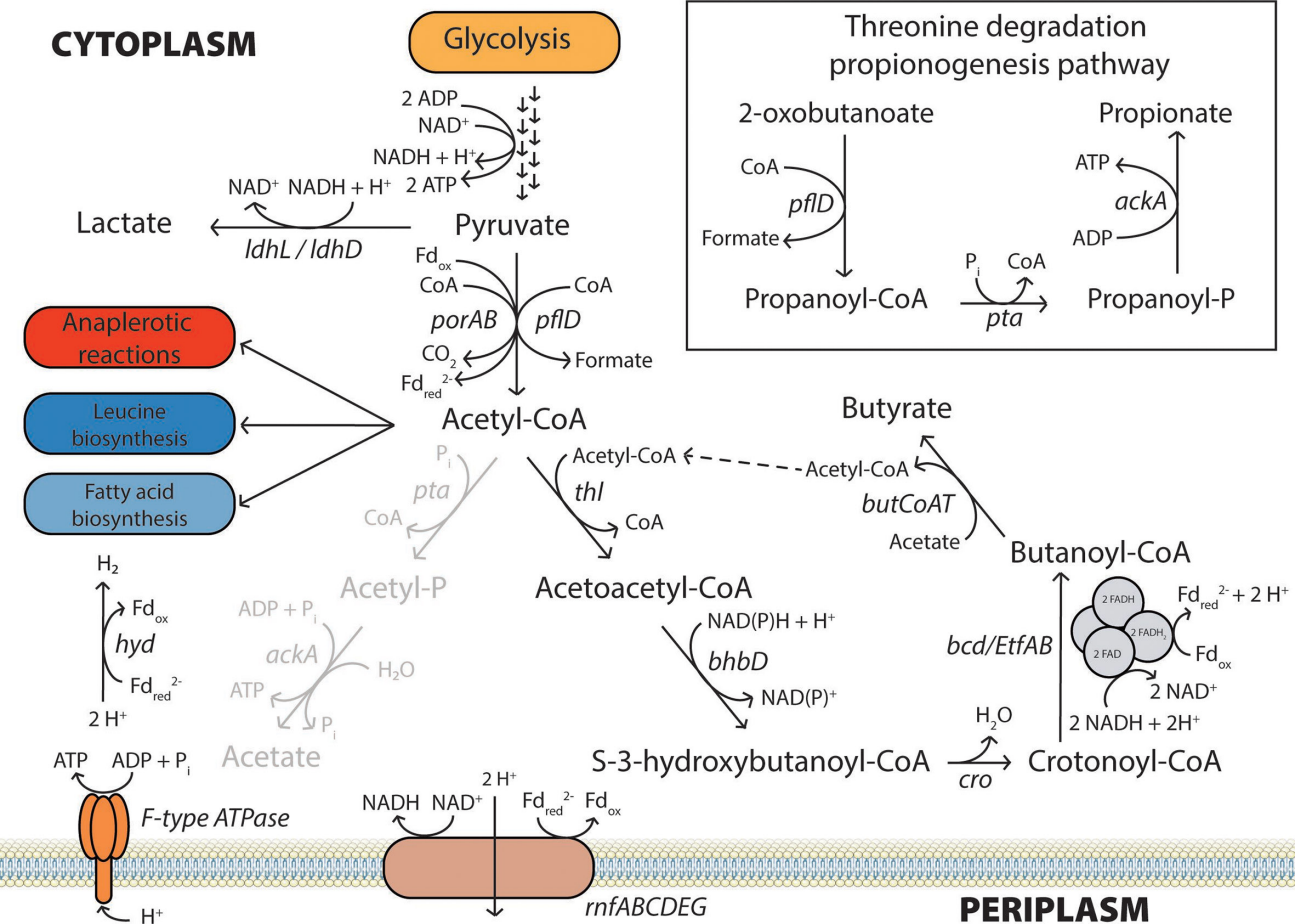
Central metabolism of *Roseburia* species, including the dominant fermentation pathways and electron transferring complexes. Although *Roseburia* can generate ATP via substrate-level phosphorylation of acetyl-phosphate to acetate, they are net consumers of acetate and thus flux through this pathway is low (indicated by the grey reaction arrows). Instead, *Roseburia* spp. appear to derive their ATP almost exclusively through ‘oxidative phosphorylation’ via an F-type ATPase via the proton gradient generated by an H^+^-translocating *rnf* complex that also recycles ferredoxins/flavodoxins. The FeFe group B hydrogenase also regenerates oxidized ferredoxin/flavodoxin while generating H_2_ (shown as H_2_ formation). Gene symbols: *ldhL/ldhD*, L-/D-lactate dehydrogenase; *porAB*, pyruvate ferredoxin oxidoreductase alpha/beta subunit; *pflD*, formate C-acetyltransferase; *pta*, phosphate acetyltransferase; *ackA*, acetate kinase; *thl*, atoB-like thiolase (acetyl-CoA acetyltransferase); *bhbD*, β-hydroxyacyl-CoA dehydrogenase; *cro*, crotonyl-CoA hydratase; *bcd*, butyryl-CoA dehydrogenase; *EtfAB*, electron transfer flavoprotein alpha and beta-subunit; *butCoAT*, butyryl-CoA : acetate-CoA transferase; *rnfABCDEG*, Na^+^/H^+^-translocating ferredoxin : NAD^+^ oxidoreductase subunits A–G; *hyd*, FeFe hydrogenase.

Although *Roseburia* spp. do not possess the traditional electron transport chain commonly used in oxidative phosphorylation, all species evaluated here have genes (*rnfABCDEG*) encoding an electron transport complex that seems to be an ancient form of electron transport chain. A similar complex is present in *Acetobacterium woodii* to translocate Na^+^, fuelled by reduced ferredoxin and generation of NADH [66]. In addition to NADH and ferredoxin cycling in this species, the F_0_F_1_ ATPase generates ATP through Na^+^ transport across the gradient generated by this Rnf complex [66, 67]. As *Roseburia* do not exhibit any butyrate kinase activity [27, 68], the generation of ATP via the gradient maintained by the Rnf complex may be vital to *Roseburia *’s survival. It is unclear whether the *Roseburia* Rnf complex translocates Na^+^ or H^+^ like others in the order Clostridiales [67, 69, 70]. In either case, the oxidation of ferredoxin (or, potentially, flavodoxins) by this complex allows *Roseburia* to regenerate the electron carriers (Fig. 3) needed for pyruvate and butyrate metabolism [69, 70]. Fermentation to pyruvate and acetyl-CoA by gut microbes produces a wide range of metabolites, including formate, lactate and short-chain fatty acids (SCFAs) [71]. Acetic acid, propionic acid and butyric acid are the most abundant SCFAs present in the human colon and have marked physiological effects on health [72]. Specifically, butyrate can be oxidized to CO_2_ by the colonocytes, which helps maintain a hypoxic epithelium and promotes energy homeostasis [54, 73].

In *Roseburia *, there are two reactions that convert pyruvate to acetyl-CoA. The first reaction incorporates a free CoA and yields CO_2_ while generating reduced ferredoxin or flavodoxin; the KEGG annotation suggests that this gene encodes a flavodoxin-utilizing enzyme, but this has yet to be experimentally determined. Many microbes typically reduce NAD^+^ to NADH in this reaction; however, anaerobes commonly utilize flavodoxin instead via the pyruvate synthase PorAB [70, 74]. Interestingly, *R. intestinalis* and *R. hominis* contain a second enzyme complex that appears to carry out the NAD^+^-to-NADH reducing reaction, as well using a tetrameric ferredoxin oxidoreductase complex. In contrast to the PorAB reaction, pyruvate formate lyase (*pflD*) carries out another acetyl-CoA-generating reaction, which produces formate directly from pyruvate. This reaction takes pyruvate and free CoA to form acetyl-CoA and formate (Fig. 3). Regulation of *pflD* is commonly carried out through a lyase-activating enzyme that lies adjacent to *pflD* in the *Roseburia* genomes [75, 76] Although these two reactions both consume pyruvate and generate acetyl-CoA, the subtle differences in electron and carbon balance may be very important to *Roseburia *’s physiology; net formate and CO_2_ production has been noted in several *Roseburia* strains [5, 7, 77]. Use of the pyruvate formate lyase may be useful when there is an overflow of pyruvate, when electron carriers can no longer be regenerated, or under conditions of iron limitation [78]. Because the *Roseburia *’s hydrogenase reaction is iron-dependent, iron deprivation has been shown to reduce the amount of butyrate and hydrogen formed while increasing lactate and formate amounts as the Rnf complex cannot regenerate ferredoxins through the formation of H_2_ [79]. Further studies will be needed to understand the regulatory schemes that determine whether *Roseburia* eliminates electrons as formate or retains them in NADH with production of CO_2_.

Lactate is also a common fermentation product of pyruvate metabolism that competes with formate and acetyl-CoA formation. Unlike *porAB* or *pflD*, lactate dehydrogenase (*ldhL*, EC 1.1.1.27) regenerates NAD^+^ by reduction of pyruvate using electrons from NADH (Fig. 3). All *Roseburia* spp. displayed strong evidence for the *ldhL* gene, and lactate has been noted as a common fermentation product in pure *Roseburia* cultures. Additionally, *R.* sp. 831b, *R. intestinalis* strains and *R. inulinivorans* strains all have two copies of *ldhL* in their genomes and are noted to make more lactate than the other species [5, 7]. *R.* sp. 831b, *R. hominis* and *R. faecis* 2789STDY5608863 also all show evidence of the *ldhD* gene, which forms d-lactate instead of l-lactate. The *R. faecis* M72 strain studied previously only has one copy of *ldh* and produced the least lactate while producing the most formate [5]. Understanding differences in pyruvate metabolism among *Roseburia* spp. in shuttling carbon and electrons and generating fermentation products may provide an insight into competition among strains and differences in potential roles in cross-feeding of the gut ecosystem [6].

While *Roseburia* do not consume lactate like other gut microbes, a net consumption of acetate has been observed for most *Roseburia* species [5, 7, 68, 80]. Despite their net consumption of acetate, *Roseburia* spp. uniformly have the genes necessary to produce acetate from acetyl-CoA and can generate ATP in the process. Although it is likely not a major *Roseburia* terminal fermentation product, acetate is important to *Roseburia *’s butyrate fermentation strategy. Rather than using butyrate kinase like other gut microbes such as *Coprococcus *, *Roseburia* use a highly active butyryl-CoA : acetate-CoA transferase [27, 68]. This is an interesting strategy because butyrate kinase yields ATP from butyryl-phosphate, while *Roseburia *’s transferase does not yield any ATP, instead generating acetyl-CoA. Five *Roseburia* strains have been shown to lack measurable butyrate kinase activity, and our genomic evidence does not support the presence of this gene except in *R. inulinivorans* LI-83, which was not among those previously tested experimentally [68]. *Roseburia *’s strategy of effectively obtaining more acetyl-CoA, however, allows *Roseburia* to make more butyrate, as each butyryl-CoA requires two acetyl-CoA in forming the precursor acetoacetyl-CoA. Duncan *et al*. [80] showed that ~85 % of the butyrate carbon was derived from extracellular acetate, ultimately, allowing *Roseburia* to recycle as much NAD^+^ from each mole of glycolysis-derived acetyl-CoA as possible. The genomic findings here are in accordance with strong prior experimental [5, 7, 27, 68, 77, 80, 81] and genomic [8, 25, 26, 82] evidence describing butyrate as the major fermentation product of *Roseburia* species.

In *Roseburia *, NAD^+^ recycling occurs in the penultimate step of butyrogenesis, where crotonoyl-CoA is converted into butyryl-CoA via butyryl-CoA dehydrogenase (*bcd*). Interestingly, its assigned KO number identified the Roseburia *bcd* as a catabolic, butyrate-consuming reaction involving the cofactor FADH_2_. In contrast, most anabolic, butyrate-forming *bcd* reactions involve the recycling of either NADH or NADPH cofactors to form butyryl-CoA. Here, it appears that *Roseburia* may use FADH_2_ instead of or in addition to NAD^+^ in an electron-transferring flavoprotein (ETF) for the formation of butyryl-CoA, based on the genome context and orthology groupings. All *Roseburia* contain a butyrogenic operon that contains an *atoB*-like thiolase (*thl*), β-hydroxybutyryl-CoA dehydrogenase (*bhbD*), *bcd* and two flavoproteins (*etfA* and *etfB*) implicated in the FADH_2_-dependent formation of butyryl-CoA [8, 82]. A similar electron-transferring flavoprotein *bcd* gene was also noted in the anaerobic clostridia, proposed by Flint and Louis [8, 83–86]. This EtfAB complex may allow *Roseburia* and other species of Clostridiales to bifurcate electrons from NADH to butyryl-CoA and ferredoxin [69, 86, 87]. Ultimately, this reaction yields the precursors for butyrogenesis, recycled NAD^+^, and reduced ferredoxin. Although TIGRFAMs TIGR01963, TIGR02280 and TIGR01751 did not match genes *bhbD*, *cro* and *bcd* above the trusted cutoff, the majority of these biosynthesis genes being located, and likely translated, together gives increased confidence that this FADH_2_-utilizing *bcd* is used here in *Roseburia* to generate, rather than degrade, butyrate. Additionally, we did not find evidence of the catabolic fatty acid oxidation pathways in which the FADH_2_-utilizing *bcd* is typically involved. As mentioned above, the final step of butyrate synthesis is carried out by a butyryl-CoA : acetate-CoA transferase rather than butyrate kinase [68], but this gene was not found in any of the previously mentioned operons. As a whole, our confidence scores for this pathway were lower than for most other pathways identified in this study, based upon our classification scheme that emphasizes TIGRFAM equivalogs. Our results argue for greater inclusion of *Lachnospiraceae* genes in seed alignments for profile HMM generation, especially as this process is central to the metabolism of this genus and its near neighbours.

The KEGG KO models also suggest that *Roseburia* may have the potential to produce propionate via a glycolysis-independent threonine degradation pathway (Fig. 3) previously noted in *Escherichia coli* [88]. Although *Roseburia* appear to possess the genetic capabilities to convert precursor compounds to propionate, there is little evidence to suggest that these species regularly ferment to propionate. In *E. coli *, the threonine degradation pathway consists of seven genes organized in a *tdcABCDEFG* operon where *tdcE* is functionally equivalent to *pflD*, and *tdcD to ackA* [88]. Although neither *pta* nor a homologue were found in this operon, *pta* can convert propionyl-CoA to propionyl-phosphate in *E. coli *. Interestingly, a threonine dehydratase similar to *tdcB*, which can generate 2-oxobutanoate by degrading threonine, is found in the same gene neighbourhood as *ackA* in all studied *Roseburia* genomes. It is unclear if its role is in isoleucine biosynthesis or if it may play a role in propionate generation, as no study has examined *Roseburia *’s metabolism solely on threonine or other amino acids as a primary carbon source. There is only one report of propionogenesis in *Roseburia: R. inulinivorans* DSM 16841, which ferments fucose to propionate via a propanediol utilization (*pdu*) operon [89]. In accordance with their findings, we were able to find and annotate this same operon, although many of the gene calls were not strongly supported using HMM-based evidence. As suggested, this is a strain-specific pathway and we could not find it in any of the other species or strains. This is not surprising, as it is rare for species to produce both propionate and butyrate [90]. Although limited propiogenesis may be a mechanism for *Roseburia* to disproportionate electrons from amino acid fermentation, future studies should investigate the ability of *Roseburia* spp. to ferment amino acids and the possible connection to propiogenesis in pure cultures. If significant amounts of propionate are produced in the gut ecosystem by amino acid fermentation by *Roseburia* or other microbes, this would be very intriguing, as the succinate, acrylate, or propanediol pathways are considered to be the common propionate synthesis pathways, with succinate being dominant [71, 90, 91]. Especially under low fluxes of dietary fibre carbohydrates, amino acid fermentation may potentially be performed by *Roseburia* spp., either concurrently with saccharolytic fermentation or after carbohydrates are exhausted, as the distal colon is known to be relatively carbohydrate-poor and to contain a higher relative abundance of peptides [92].

Interestingly, in addition to fermentation, some *Roseburia* spp. may be able to disproportionate electrons using sulfate as an electron acceptor. All studied *R. faecis* and *R. intestinalis* strains contained a putative operon containing genes for the ABC sulfate transporter *cysPUWA*, the sulfate adenlylyltransferase *cysND,* which generates adenosine-5′-phosphosulfate (APS) [93], and the APS reductase *aprAB*, which reduces APS to sulfite while oxidizing a reduced electron carrier (typically, NADH) [94]. The eventual fate of produced sulfite in *Roseburia* is unclear, as all examined genomes lack the dissimilatory sulfite reductase *dsrAB* that reduces sulfite to sulfide.

#### Amino acid biosynthetic pathways

Protein synthesis requires biosynthesis or uptake of all 20 canonical amino acids. Amino acids can be synthesized *de novo* from organic precursors, including intermediates of glycolysis and the TCA cycle, such as pyruvate and α-ketoglutarate, by amination, which requires exogenous nitrogen. In the case of *Roseburia *, luminal nitrogen in the colon can be derived exogenously from the host’s diet, endogenously from sloughed intestinal cells, or through nitrogen cycling in the intestine [95]. All *Roseburia* spp. are capable of accessing inorganic nitrogen as ammonium for amino acid biosynthesis, synthesizing glutamate from α-ketoglutarate via glutamate synthase (also termed glutamine oxoglutarate transaminase, or GOGAT) and glutamine via a type 1 glutamine synthetase (*glnA*). However, only *R. intestinalis* species appear to have the potential to use urea as a nitrogen source via the biotin-requiring urea carboxylase encoded by the *uca* operon; appreciable evidence for the presence of ureases was not detected in any *Roseburia* species. As urea is excreted into the colonic lumen through the epithelium, it represents a potential competitive advantage for *R. intestinalis* under conditions of strong competition for ammonium (for example, during highly saccharolytic conditions).

*Roseburia* species appear to be able to synthesize nearly all of their own amino acids *de novo*. This result was somewhat surprising given the high organic carbon and nitrogen content of colon luminal contents; however, excreted amino acids have long been thought to be largely bound in proteins [96] and faecal metagenomes reveal significant enrichments in amino acid biosynthetic genes relative to all sequenced bacterial genomes in KEGG [97]. This may indicate either low bioavailability of free amino acids in the colon or fierce competition for amino acids among organisms. All of the *Roseburia* genomes we examined possessed complete proline biosynthesis pathways from glutamate and arginine biosynthesis pathways via citrulline and aspartate. However, all appeared to lack arginases and, therefore, possessed incomplete arginine cycles. All species of the genus *Roseburia* encode genes for the biosynthesis of aspartate from oxaloacetate and asparagine from aspartate. The entirety of the lysine biosynthesis pathway converting aspartate to lysine via the dehydrogenase branch is present throughout the genus. However, all three strains of *R. inulinivorans* contain genes suggesting the presence of an additional alternative succinylation-dependent synthesis branch.

With respect to serine biogenesis, all members of the genus encode the first two genes in the phosphorylation pathway, D-3-phosphoglycerate dehydrogenase (*serA*) and phosphoserine aminotransferase (*serC*), but appear to lack phosphoserine phosphatase (*serB*). These serine biosynthesis genes are located within a predicted operon with genes encoding enzymes that catalyze the first committed steps of branched chain amino acid (BCAA) synthesis from pyruvate (acetolactate synthase, acetolactate reductoisomerase and 2,3-dihydroxyisovalerate dehydratase), which is conserved across all *Roseburia* genomes. Notably, only the large catalytic subunit of acetolactate dehydrogenase is present within the operon; in *E. coli* the large subunit alone is catalytically active, though at a slower rate, and is insensitive to feedback inhibition [98]. Both subunits of acetolactate synthase are present in a separate operon in all examined *Roseburia* genomes, suggesting the hypothesis that this combined serine-BCAA synthesis operon responds to low amino acid concentrations to increase the flux of pyruvate into serine and BCAAs. Genomes containing *serAC* but lacking *serB* are common within Firmicutes [99], which has led to predictions that many members of this phylum are serine auxotrophs [100]. Some members of *Dehalobacter* have been shown to escape a lack of *serB* by synthesis of serine from glycine via serine hydroxymethyltransferase [99], which all the *Roseburia* genomes we analysed also possess. However, if this strategy is broadly used across Firmicutes to synthesize serine, the reason for the frequent retention of *serA* and *serC* genes in these genomes is unclear. Recently, homoserine kinase (*thrH*) has been shown to also catalyze the same dephosphorylation reaction as *serB* [101]; TIGRFAM and FIGfam annotations have identified a putative *thrH* gene for all *Roseburia* genomes except *R. hominis* and *R*. sp. 831b. The discovery of alternative serine synthetic strategies in Firmicutes may help resolve the paradox that, although it is among the least expensive amino acids to synthesize [99], predicted auxotrophy for serine is widespread based upon our present understanding of possible serine biosynthetic pathways.

From serine, all *Roseburia* genomes possess biosynthesis genes for glycine via glycine hydroxymethlyransferase (*glyA*). This enzyme also catalyzes the interconversion of glycine and threonine using acetaldehyde as a substrate. This is the sole threonine biosynthetic path in *R. hominis* and *R*. sp. 831b, which lacked evidence for homoserine kinase. Cysteine is produced by direct sulfurylation with sulfide by the CysEK complex. FIGfam predictions for multiple aminotransferases were also identified for all species. Of note, *R. intestinalis* genomes appeared to lack the *murI* glutamate racemase, required to interconvert d- and l-glutamate. Although l-glutamate is used in protein synthesis, d-glutamate is required in many organisms for peptidoglycan biosynthesis, which may suggest either a requirement for exogenous d-glutamate or an altered cell wall structure in this *Roseburia* species [102].

The genes required for biosynthesis of hydrophobic amino acids are present in almost all species of *Roseburia *. With respect to branched-chain amino acids, evidence for the biosynthesis of leucine, valine and isoleucine is present for all examined species of *Roseburia* with moderate to high confidence. However, the evidence for the direct biosynthesis of d-alanine was weak for common biosynthetic routes (i.e. from pyruvate or aspartate), lacking TIGRFAM and KO identifications. Notably, *R*. sp. 831b was the only species displaying KO evidence for *alaA*, an alanine transaminase, although biosynthesis via cysteine desulfurase or through racemization of alanine was well supported. Due to the lack of high-confidence predictions for d-alanine biosynthesis, we also annotated genomes at the protein domain level (i.e. Pfams) for every *Roseburia* species herein. Notably, Pfams provided evidence for an alanine symporter (Table S6); alanine glyoxylate aminotransferase, alanine dehydrogenase and a class IV aminotransferase were identified for all species. Additionally, some species of *Roseburia* showed significant Pfam hits for PF02261 (aspartate decarboxylase). Apart from *R. inulinivorans* DSM 16841, which lacked any evidence for tryptophan biosynthesis, all *Roseburia* genomes displayed complete pathways for aromatic amino acid biosynthesis.

**Fig. 4.**
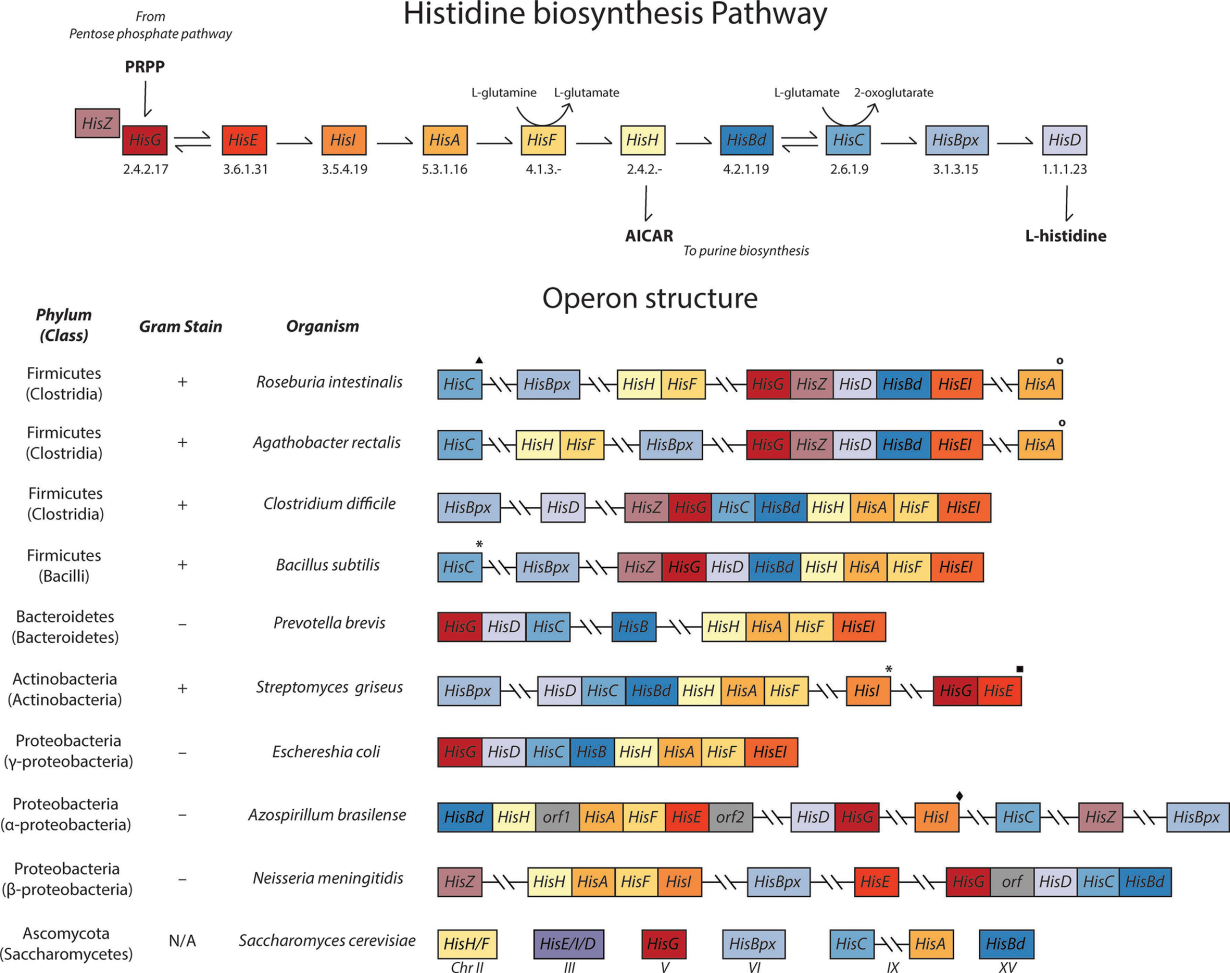
Histidine biosynthetic pathway and operon structure. The histidine biosynthetic pathway (top) not only synthesizes l-histidine from 5-phospho-d-ribose α-1-pyrophosphate (PRPP), but also the precursor of purine biosynthesis, 5-aminoimidazole-4-carboxamide ribonucleotide (AICAR). Across the bacterial kingdom, the histidine operon structure is paraphyletic, displaying similar organization among closely related species, while more distant ancestors have varying organizations. To demonstrate these similarities, the operon organizations of various representative microbes across different phyla and classes are shown (bottom). Gene names: *HisA*, phosphoribosylformimino-5-aminoimidazole carboxamide ribotide isomerase; *HisBd*, imidazoleglycerol-phosphate dehydratase; *HisBpx*, histidinol-phosphatase; *HisC*, histidinol-phosphate aminotransferase; *HisD*, histidinol dehydrogenase; *HisF*, imidazole glycerol-phosphate synthase cyclase subunit; *HisG*, ATP phosphoribosyltransferase; *HisH*, glutamine amidotransferase; *HisEI*, phosphoribosyl ATP pyrophosphohydrolase/phosphoribosyl-AMP cyclohydrolase; *HisZ*, ATP phosphoribosyltransferase regulatory subunit. The chromosome number where the gene is located is displayed for eukaryotes. Genes in the other biosynthetic operons are denoted as follows: ▲, tyrosine/phenylalanine biosynthesis; *, tryptophan biosynthesis; ■, riboflavin biosynthesis; ○, glutamine biosynthesis; ♦, cystine biosynthesis

All studied genomes from *Roseburia* are predicted to contain the genes required for histidine biosynthesis at high confidence. Histidine biosynthesis is an energetically expensive task [103] and is often tightly regulated within a single operon (Fig. 4). However, unlike the *E. coli* histidine (*his*) operon that contains all the pathway genes in one locus, the majority of *Roseburia *’s *his* genes are divided among multiple loci (Fig. 4). Specifically, *hisG*, *hisZ*, *hisD*, *hisBd* and *hisEI* are present in a single predicted operon in every subspecies, and the genes *hisF* and *hisH* are located in a separate predicted operon across all genomes. The *hisBpx* gene is not located within a predicted operon. Finally, *hisA* and *hisC* are not located in either of the other *his* predicted operons, but are housed with glutamine synthetase and aromatic aminotransferase, respectively.

Previously, a partial *his* operon has been observed in the Gram-negative alpha-proteobacterium *Azospirillum brasilense* [104] and Gram-positive *Bacillus subtilis* [105]. While the *E. coli his* operon structure is well known, the organization of *his* genes in clusters/operons is variable among distantly related microbes. As shown in Fig. 4, similar operon organization among closely related species is displayed across Firmicutes, while organization varies considerably in more distant genomes. The organizational diversity of the *his* operon suggests that various separations, fusions and relocations of these genes within the genome have occurred (multiple lateral transfers), emphasizing that there are multiple ways to optimize control of histidine biosynthesis for different environments [106, 107].

In eukaryotes, such as *S. cerevisiae*, the *his* genes are not located in clusters, but gene fusions (with respect to the gene organization frequently observed in bacteria) are common. Gene fusions of *hisE*/*I*/*D* and *hisF*/*H* have been discovered and it has been suggested that they help control flux through the pathways with substrate tunnelling [104, 106]. Although *Roseburia* spp. do not have a *hisF/H* fusion gene, the *hisF* and *hisH* genes are organized together in a predicted operon that may, in a similar way, increase the efficiency with which flux is regulated through the pathway. Similar to *S. cerevisiae*, *Roseburia *’s *hisE/I* fusions may be a product of convergent evolution or horizontal gene transfer and are thought to allow efficient biosynthesis using substrate tunnelling mechanisms [108].

Gene organization that may promote efficient regulation of flux into histidine biosynthesis and/or increase the efficiency of reaction mechanisms is not surprising, given the high energetic cost, scarcity of nutrients in the colon and importance of both His and its byproducts [109]. To further enhance *his* operon regulation, *Roseburia *’s *hisG* protein likely requires an additional catalytic polypeptide, HisZ, to initiate biosynthesis from phosphoribosyl diphosphate [110]. HisZ is allosterically regulated by ATP and histidine, which provides feedback inhibition [111]. Colocalization in a predicted operon with glutamine synthetase suggests that histidine and glutamine biosynthesis may be co-regulated; this type of coregulation with serine biosynthesis was also suggested in the *his* operon of the anaerobe *Streptococcus mutans* UA159 [112]. Additionally, an important byproduct of the HisF–HisH reaction is 5-aminoimidazole-4-carboxamide ribonucleotide (AICAR), which is used in purine biosynthesis, and thus may require expression independently of the rest of the *his* genes under conditions where purines, but not histidine, are required. Further experiments to evaluate how *Roseburia* regulates these two pathways will be required to predict the function of this gene organization.

#### Biosynthesis of B vitamins

**Fig. 5.**
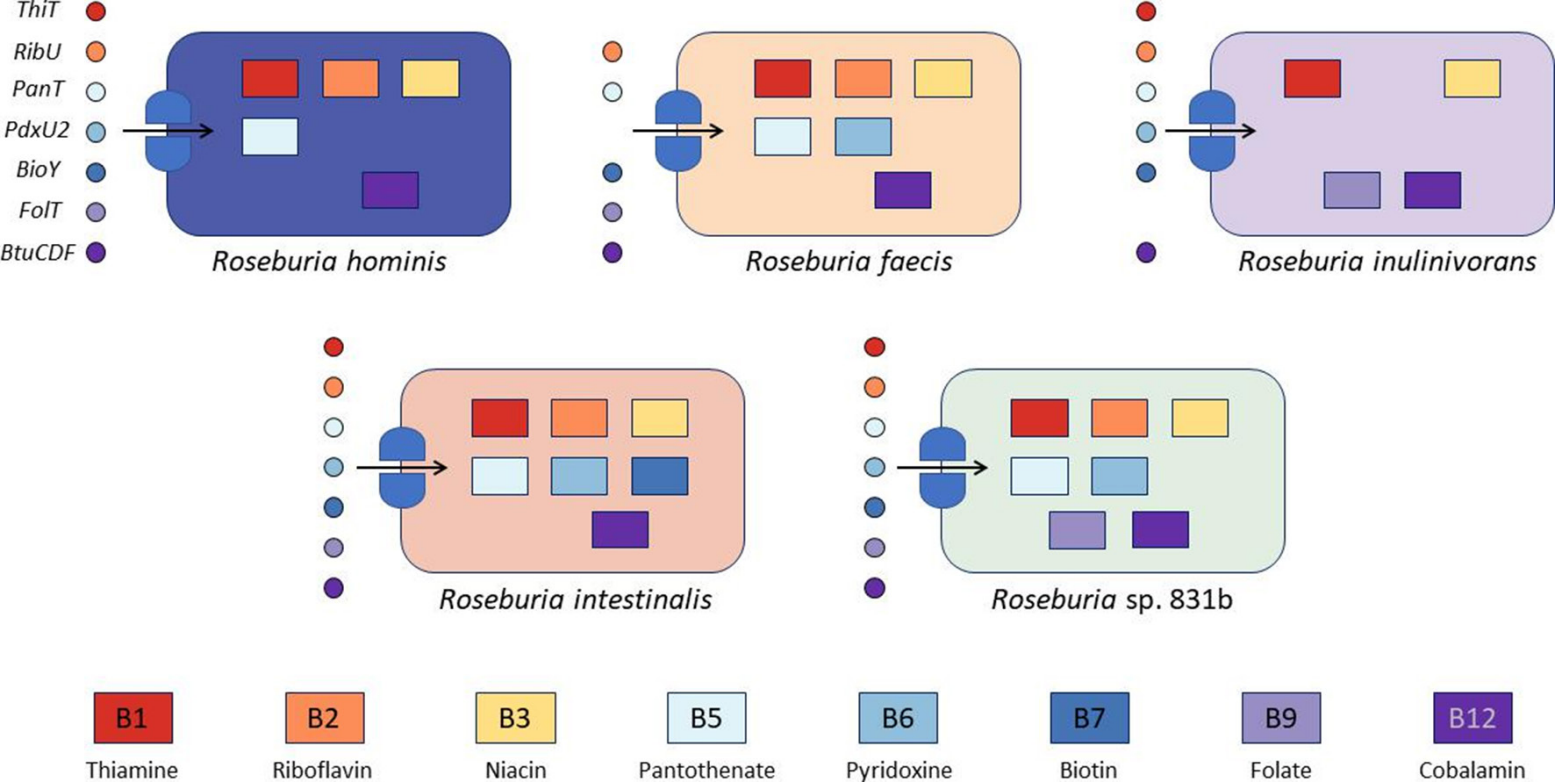
Vitamin synthesis and transport in *Roseburia* species. Rectangles represent biosynthetic pathways and circles represent transporters that were predicted by the species indicated. Each shape is coloured to represents a particular B vitamin that we predict can be synthesized or transported. The *ThiT*, *RibU*, *PanT*, *PdxU2*, *BioY* and *FolT* genes are energy coupling factor (ECF)–type transporters for thiamine, riboflavin, pantothenate, pyridoxine, biotin and folate, respectively, while *BtuCDF* is an ATP-binding cassette (ABC) transporter with a substrate-specific domain for cobalamin transport.

B vitamins are essential cofactors for many enzymes across the tree of life, and are thought to exert strong influence over microbial ecology in multiple environments, including the gut [18, 21, 113]. In humans, B vitamin requirements are met through dietary consumption, but may also be produced by the gut microbiota. We sought to determine how *Roseburia* spp. meet their B vitamin requirements. Our results show that most *Roseburia* spp. have the ability to either synthesize or transport all of the B vitamins (Fig. 5). We predict that none of the examined *Roseburia* spp. can synthesize biotin, but all can synthesize thiamine, pyridoxine, folate and cobalamin. Synthesis of riboflavin, niacin and pantothenate varied at the species level, as did salvage transporters for folate, pyridoxine and thiamine.

Despite their shared synthesis capabilities, we found genes for alternative synthesis strategies among *Roseburia* species that may minimize competition for these public goods [18, 19]. In the case of thiamine*,* which is required for diverse catabolic reactions of sugars and amino acids, we did not detect the *thiC* gene within *R. inulinivorans* strains, which is the first gene required in the pathway. However, analysis revealed with high confidence the transporter gene *cytX* in all *R. inulinivorans* genomes that carries hydroxymethylpyrimidine [114], which can then be converted into a precursor for thiamine with the bifunctional enzyme *thiD* . Additionally, we found evidence for a thiamine energy coupling factor (ECF) transporter [115], *thiT*, in all species except *R. faecis *.

In general, we also found strong evidence for niacin synthesis via aspartate and tryptophan in all *Roseburia* spp. Additionally, all genomes exhibited *pncB*, which catalyzes the one-step production of nicotinate d-ribonucleotide from nicotinic acid. However, all genomes except *R*. sp. 831b*, R. hominis* and *R. faecis* also displayed strong evidence for *surE*, which catalyzes the synthesis of both nicotinate d-ribonucleotide and nicotinamide d-ribonucleotide from their cognate nucleoside precursors. These differences may suggest increased biosynthetic flexibility for nicotinamide in these species. All *Roseburia* spp. except 831b also possess the ability to interconvert nicotinamide and nicotinic acid via PncA. Interestingly, we did not detect the presence of any niacin transporters [116], suggesting that *de novo* synthesis is the sole route of NAD^+^ production for the genus *Roseburia *.

*R. inulinivorans* apparently lacked the ability to biosynthesize multiple B vitamins common to other *Roseburia* species. All of the genomes we examined are predicted to contain riboflavin (vitamin B_2_) biosynthesis genes, the precursor for the synthesis of FMN and FAD, with high confidence. Consistent with other studies [117, 118], evidence for the uracil transporter *pyrP* was not observed for any species. *R. inulinivorans *, however, does possess *ribF*, which allows it to derive FMN and FAD from riboflavin. Rather than synthesizing riboflavin, we propose that *R. inulinivorans* strains salvage riboflavin using the riboflavin ECF transporter *ribU*. Similarly, all genomes save those within *R. inulinivorans* are able to synthesize pantothenic acid (vitamin B_5_) from either aspartic acid or β-alanine using the *panC* and *panD* genes. FIGfam predictions also included a vitamin B_5_ ECF transporter (*panT*) that was present in all of the *Roseburia* genomes (inclusive of *R. inulinivorans *). The loss of genes required for cofactor synthesis may suggest a niche for *R. inulinivorans* in regions of the colon where these vitamins are more available. This is consistent with its ability to consume fast-fermenting oligosaccharides such as inulin, which ferment largely in the cecum and ascending colon, or over time periods where dietary vitamin intake is higher or competition lower (e.g. when intestinal transit is faster).

In contrast, we found strong evidence for synthesis of folate, which is required for one-carbon metabolism from purines, in *R. inulinivorans* genomes but not in other *Roseburia* spp. Interestingly, in *R. inulinivorans* genomes the genes encoding the first and last dedicated steps in folate biosynthesis (*folE* and *folC*, respectively) from GTP are clustered in a predicted operon; the rest of the biosynthetic genes reside together in a likely second operon. This curious arrangement may permit increased regulatory control over folate biosynthesis by this species. Although we did not find evidence of biosynthesis in any of the other *Roseburia* species, all other species possess a putative *folT* folate ECF transporter.

Acquisition strategies for vitamin B_6_ (pyridoxine) also varied across the genus, dividing along synthesizer (using the yeast-type synthesis pathway encoded by *pdxST*) and salvager (*pdxKYH*) strategies. With high confidence, all *R. intestinalis* strains and *R*. sp. 831b contained the yeast-type synthesis genes, in which either the PdxT/PdxS complex forms pyridoxine 5′-phosphate (PLP) from l-glutamine or PdxS generates pyridoxal phosphate from either l-ribulose-5-phosphate or glyceraldehyde-3-phosphate. Lower-confidence predictions asserted the presence of *pdxST* in *R. faecis* 2789STDY5608863, but we did not observe any evidence for these genes in *R. faecis* M72, *R. hominis* and *R. inulinivorans* genomes. However, we only observed pyridoxine transporter *pdxKYH* genes with moderate confidence in *R. faecis* spp. and *R*. sp. 831b. *R. inulinivorans* genomes lacked pyridoxine synthesis, but we found strong evidence for the pyridoxamine ECF transporter gene, *pdxU2*, in all *Roseburia* species except for *R. faecis *.

Biotin is required as a cofactor central to carboxyl group transfer [119] and is involved in pyruvate interconversion with oxaloacetate as well as amino acid, fatty acid and urea metabolism (via urea carboxylase), among other pathways [18]. Biotin synthesis is a tightly regulated and energetically costly process [120], and we only found evidence for synthesis from pimelate thioester in the *R. intestinalis* genomes studied here. However, these genomes still lacked genes for the synthesis of pimelate from the fatty acid synthesis (Fab) pathway, as well as the *bioF* and *bioW* genes, which are required for the production of 8-amino-7-oxononanoate from pimelate. *R. intestinalis* was the only species that was able to form biotin from this precursor via BioA and BioD, although the transporter to salvage this compound is unknown. Along with *R. intestinalis,* all studied *R. inulinivorans* genomes and *R. faecis* M72 also possessed the *bioB* biotin synthase gene that allows synthesis of biotin from dethiobiotin. Our predictions suggest that, due to the energetic costs of synthesis, *Roseburia* spp. use salvage strategies to obtain free biotin in the distal colon rather than synthesize it, as biotin synthesis pathways are rare within the phylum Firmicutes [118]. All studied genomes displayed evidence of the *bioY* biotin transporter gene with high confidence, which is usually accompanied by other components of the ECF transporter [116]. However, BioY has been observed to transport biotin without additional components in *E. coli* [121]. The presence of different biotin salvage pathways among *Roseburia* species suggests that these organisms may reduce head-to-head competition for biotin by transporting different precursors [18]. *R. intestinalis* may have increased demand for biotin due to its biotin-requiring urea carboxylase; these genomes correspondingly contain the most elaborate biotin salvage pathway.

Finally, we found evidence for all genes in the anaerobic cobalamin synthesis pathway from siroheme except *cobR* in all *Roseburia* genomes. Cobalamin, known as vitamin B_12_, is critical for some ribonucleotide reductases and methionine synthases, and is thus required for dNTP and methionine production by some species [21]. Furthermore, B_12_ is an essential cofactor in propiogenesis strategies [18], which may impact on SCFA output in the colon. Although cobalt is inserted early in the anaerobic biosynthetic pathway [122, 123], cobalt reductase (CobR) reduces Co^2+^ to Co^+^ in the final steps of cobalamin synthesis in both pathways [124]. Since we predict the presence of all other necessary vitamin B_12_ synthesis genes in all *Roseburia* spp., we hypothesize that either CobR is not utilized in the anaerobic pathway, as suggested in Magnusdottir *et al*. [118], another gene performs this reduction step, or that Co^+^ availability is high enough in the reducing environment of the colon to meet the very small amounts required. Like most other B vitamins, we found evidence for a B_12_ transporter; *btuCDF* encodes an ABC transporter with ATPase and permease domains (C and D) and a B_12_-binding domain (F) [125, 126]. The BtuCDF complex is not an ECF transporter and is distinct from the recently discovered *cbrT* B_12_ ECF transporter [127].

**Fig. 6.**
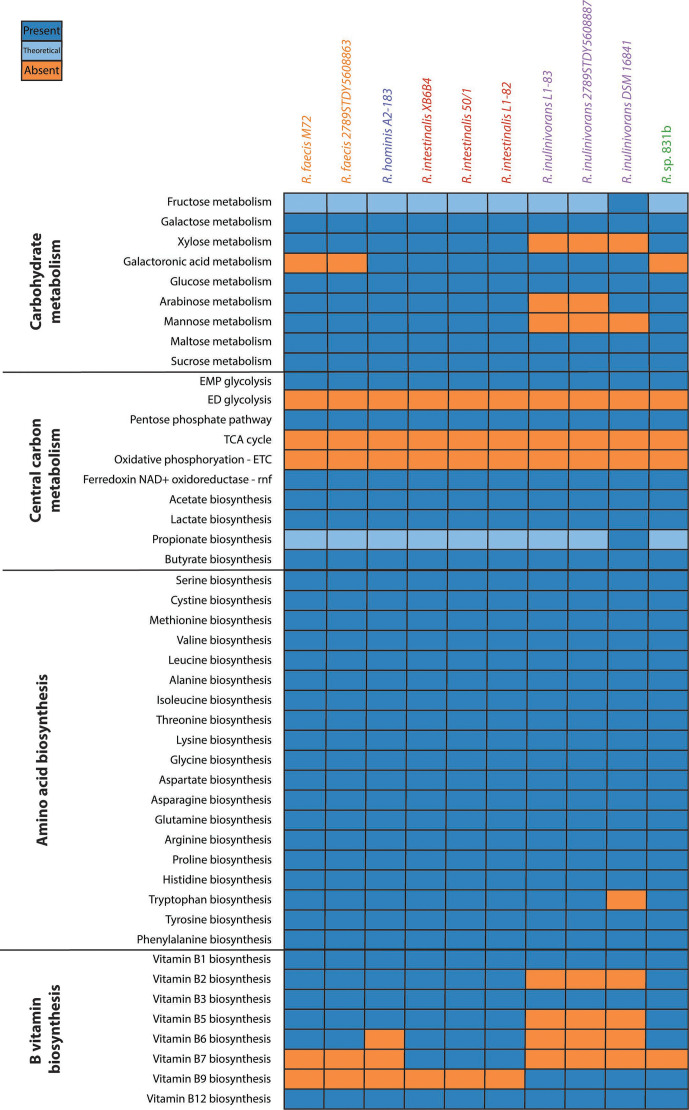
Overview of *Roseburia *’s metabolic and biosynthetic capabilities based on this analysis.

Although much of the influence over *Roseburia* spp. ecology in the gut is thought to stem from divergent carbon source preferences [24, 57], differences in the vitamin biosynthetic and salvage pathways of *Roseburia* species may underscore a different set of genome-encoded ecological strategies in which different *Roseburia* species may conditionally exhibit increased fitness. As inferred from ecological modelling [128] and observed in recent human microbiome studies, data and theory suggest that competition for resources is strongest between members of the same genus [129]. Salvage may dominate *Roseburia* vitamin acquisition in the gut ecosystem; we found evidence of transporters for all vitamins except niacin (Fig. 5). In some cases, for example, we also found evidence for multiple cobalamin transporters, which is consistent with the hypothesis that gut microbes may specialize in salvage of different precursors for the same vitamins, maximizing diversity and minimizing competition [18, 21]. Moreover, it has been proposed that the gut microbes compete with the host for diet-derived cobalamin and related corrinoids [21]; functional degeneracy in transporters may allow organisms dependent upon vitamin salvage multiple avenues to meet their cofactor needs with respect to dynamic concentrations and conditions [20]. This may help explain our observation that some species, such as *R. faecis* and *R. inulinivorans *, do not possess the same biosynthetic capabilities for energetically expensive cofactors as their cousins. These organisms may be adapted for transient conditions of relatively high vitamin availability. Magnustottir *et. al* analysed the genomes of several human gut microbes for B vitamin synthesis capabilities [118], identifying several ‘pattern pairs’ – patterns in the presence/absence of synthesis genes for each B vitamin – in their selected microbes (Fig. 6). We searched for these patterns in our *Roseburia* species but did not find evidence for them, highlighting the need for further research to determine the functional roles and competitive strategies gut commensals (including *Roseburia *) employ to maintain fitness. Our results suggest that the role of biosynthetic capabilities in determining ecological outcomes in the gut in particular should be more extensively investigated.

## Data Bibliography

1. Browne HP, Forster SC, Anonye BO, Kumar N, Neville BA, Stares MD, Goulding D, Lawley TD. Culturing of 'unculturable' human microbiota reveals novel taxa and extensive sporulation. *Nature*, 2016 May; 533(7604):543-546. DOI: 10.1038/nature17645

2. Trachsel J, Bayles DO, Looft T, Levine UY, Allen HK. Function and Phylogeny of Bacterial Butyryl Coenzyme A:Acetate Transferases and Their Diversity in the Proximal Colon of Swine. *Appl Environ Microbiol*, 2016 Nov; 82(22):6788-6798. DOI: 10.1128/AEM.02307-16

3. Duncan SH, Hold GL, Barcenilla A, Stewart CS, Flint HJ. Roseburia intestinalis sp. nov., a novel saccharolytic, butyrate-producing bacterium from human faeces. Int. J. Mol. Evol. Microbiol. 2002 Sep; 52(5):1615-1620. DOI: 10.1099/00207713-52-5-1615

4. Duncan SH, Aminov RI, Scott KP, Louis P, Stanton TB, and Flint HJ. Proposal of Roseburia faecis sp. nov., Roseburia hominis sp. nov. and Roseburia inulinivorans sp. nov., based on isolates from human faeces. Int. J. Syst. Evol. Microbiol. 2006 Oct; 56(10):2437-2441. DOI: 10.1099/ijs.0.64098-0

5. Pajon A, Turner K, Parkhill J, Bernalier A. **The genome sequence of Roseburia intestinalis XB6B4.** metaHIT consortium -- http://www.metahit.eu/ Submitted MAR-2010 to the EMBL/GenBank/DDBJ databases*.* GCA_000210655.1.

6. Pajon A, Turner K, Parkhill J, Duncan S, Flint H. **The genome sequence of Roseburia intestinalis M50/1.** metaHIT consortium -- http://www.metahit.eu/ Submitted MAR-2010 to the EMBL/GenBank/DDBJ databases. GCA_000209995.1.

7. Sudarsanam P, Ley R, Gurunge J, Turnbaugh PJ, Mahowald M, Liep D, Gordon J. **Draft genome sequence of Roseburia inulinivorans (DSM 16841).** Submitted MAR-2009 to the EMBL/GenBank/DDBJ databases*.* GCA_000174195.1.

## Supplementary Data

Supplementary material 1

Supplementary material 2
