# Supplementary material for: Comparative genomics of the genus Roseburia reveals divergent biosynthetic pathways that may influence colonic competition among species
Source: Microb Genom. 2020 Jun 26;6(7):mgen000399. doi: 10.1099/mgen.0.000399 (PMC7478625; doi:10.1099/mgen.0.000399)
Supplement: Supplementary material 1 [file mgen-6-399-s001.pdf]

Comparative genomics of genus *Roseburia* metabolism reveals divergent biosynthetic pathways that may influence colonic competition among species

Ethan T Hillman[1,2], Ariangela J Kozik[2,3,†], Casey Hooker[1], John L Burnett[4], Yoojung Heo[5], Violet A Kiesel[6], Clayton J Nevins[5,‡], Jordan MKI Oshiro[6], Melissa Marie Robins[1], Riya D Thakkar[4,7], Sophie Tongyu Wu[4], Stephen R Lindemann[2,4,7]

1 - Department of Agricultural and Biological Engineering, Purdue University

2 - Purdue University Interdisciplinary Life Science Program (PULSe), Purdue University

3 - Department of Comparative Pathobiology, Purdue University

4 - Department of Food Science, Purdue University

5 - Department of Agronomy, Purdue University

6 - Department of Nutrition Science, Purdue University

7 - Whistler Center for Carbohydrate Research, Purdue University

West Lafayette, IN 47907, United States of America

† Current address: Division of Pulmonary and Critical Care Medicine, Department of Internal Medicine, University of Michigan 1500 East Medical Center Dr. 3916A Taubman Center, SPC 5360 Ann Arbor MI 48109

‡ Current address: University of Florida, Department of Soil and Water Sciences 1692 McCarty Dr. Gainesville, FL 32603

Supplemental Figures

Figure S1 – Fully expanded 16S rRNA gene phylogenetic tree

Figure S2 – Fully expanded 18 concatenated gene phylogenetic tree

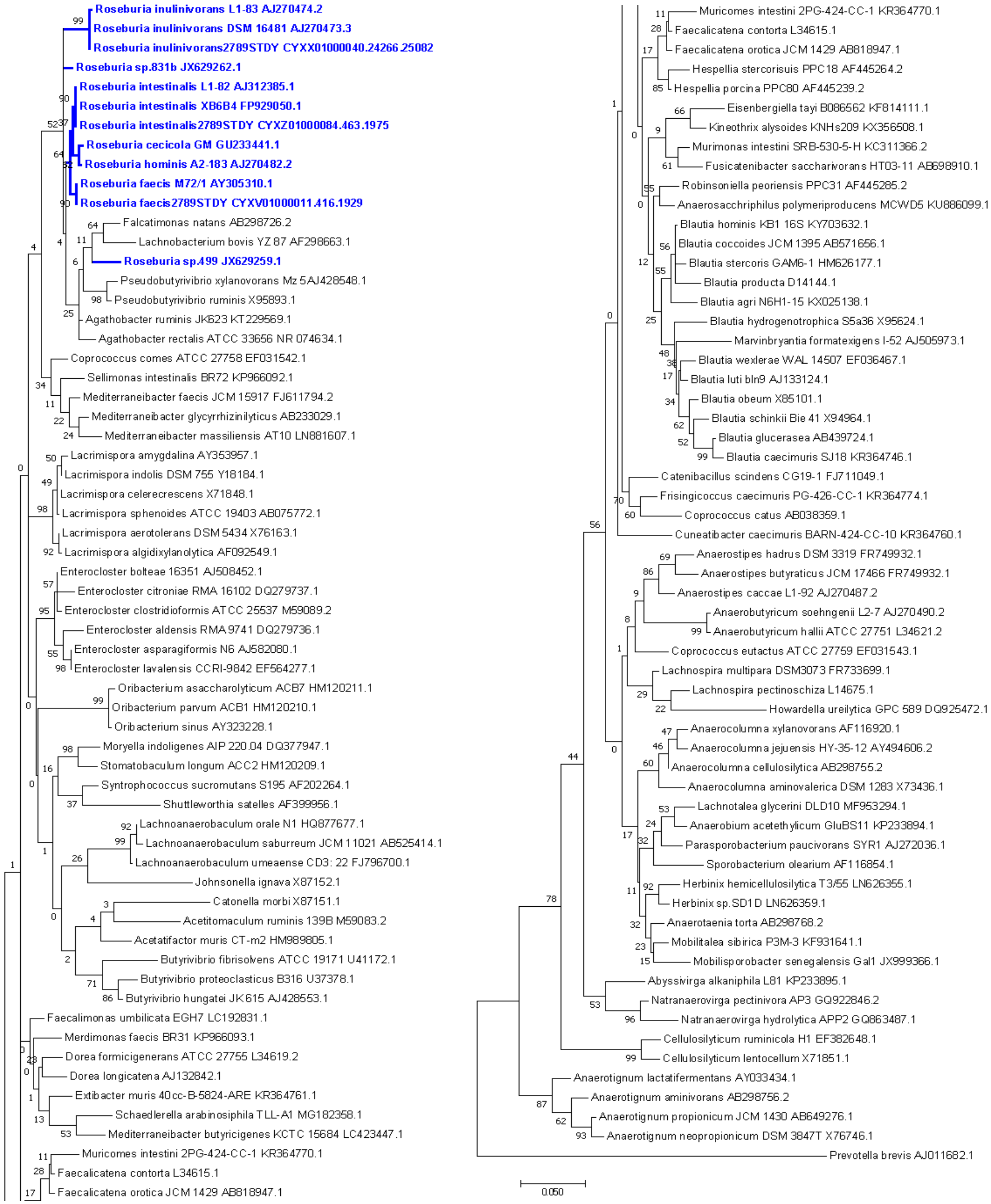

Figure S1 – Fully expanded phylogenetic trees including accession numbers of the *Lachnospiraceae* family. Full-length 16S rRNA gene maximum likelihood tree from 1000 bootstrap replicates is shown (Bootstrap scores are reported).

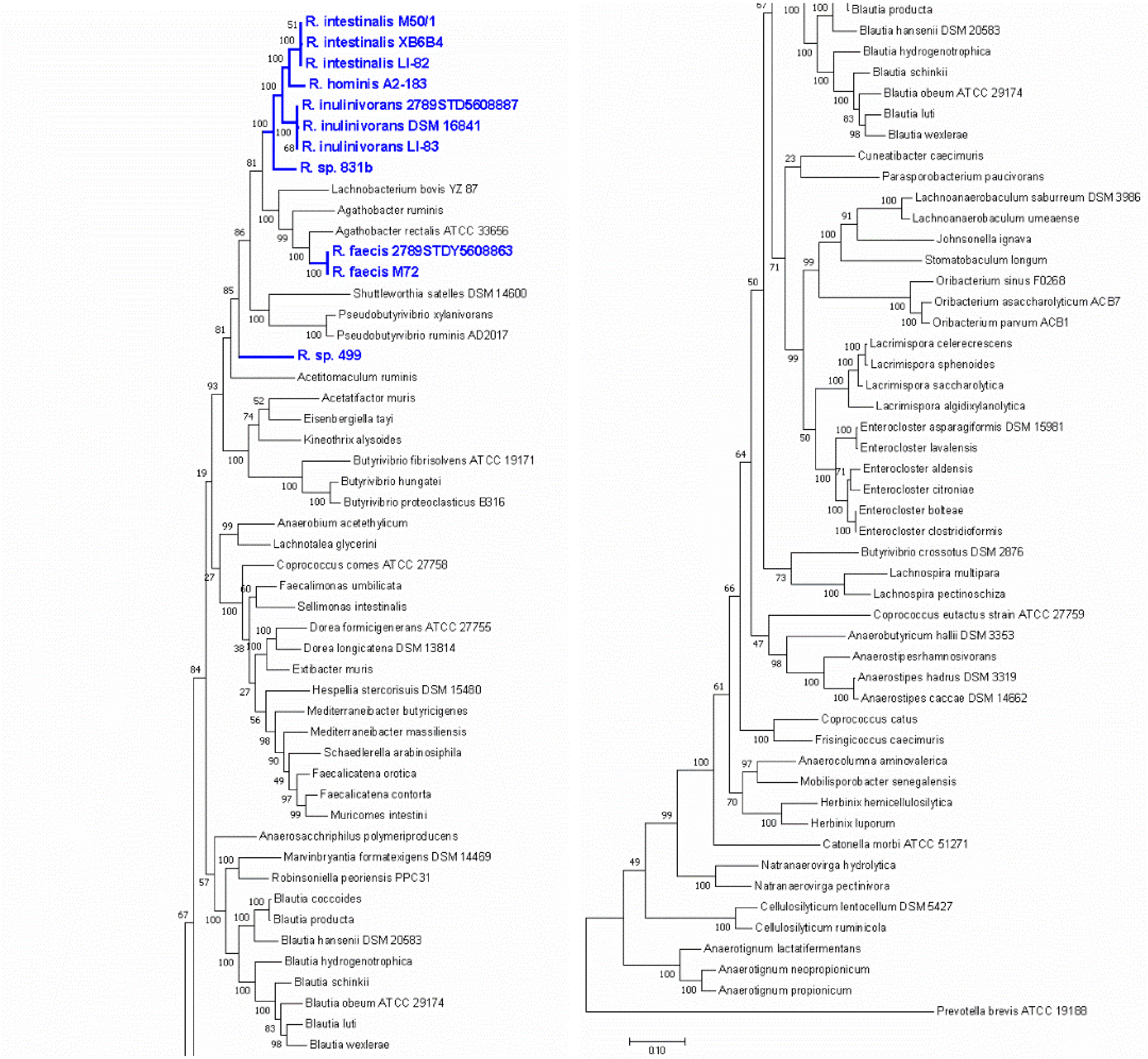

Figure S2 – Fully expanded 18 concatenated gene phylogenetic tree of the *Lachnospiraceae* family. Maximum likelihood tree from 100 bootstrap replicates is shown (Bootstrap scores are reported).
